# A review of the cleptoparasitic bee genus *Townsendiella* (Apidae, Nomadinae, Townsendiellini), with the description of a new species from Pinnacles National Park

**DOI:** 10.3897/zookeys.546.6443

**Published:** 2015-12-16

**Authors:** Michael C. Orr, Terry L. Griswold

**Affiliations:** 1 Biology Department, Utah State University, Logan, Utah, 84322, USA; 2 USDA-ARS Pollinating Insects Research Unit, Logan, Utah, 84322-5310, USA

**Keywords:** Apoidea, taxonomy, new species, cleptoparasitism

## Abstract

The cleptoparasitic bee genus *Townsendiella* Crawford (Nomadinae, Townsendiellini) is a rare group restricted to the southwestern United States and adjacent Mexico, whose taxonomy and biology remain poorly known. This paper describes *Townsendiella ensifera* **sp. n.**, the first known record of this genus from Pinnacles National Park. A key to the species of the genus is provided. Several potential areas of future research are also discussed.

## Introduction

*Townsendiella* Crawford, the only member of the tribe Townsendiellini, are one of several (e.g., *Hexepeolus* Linsley and Michener, *Rhopalolemma* Roig-Alsina) rarely collected cleptoparasitic apid bees endemic to the southwestern United States and adjacent northern Mexico (Michener 2007). Originally monobasic (Crawford 1916), Linsley (1943) reviewed the genus and divided it into three monotypic subgenera: *Townsendiella* Crawford, 1916 (type species: *Townsendiella pulchra* Crawford, 1916), *Xeropasites* Linsley, 1943 (type species: *Townsendiella rufiventris* Linsley, 1942), and *Eremopasites* Linsley, 1943 (type species: *Townsendiella californica* Michener, 1936). Michener (2000) later synonymized *Xeropasites* and *Eremopasites* with *Townsendiella* s. str. The genus has recently been included in phylogenetic studies, where it is apparently closely related to two other clades of Nomadinae: ((Townsendiellini+Neolarrini)+Biastini) (Cardinal and Danforth 2013, Payne 2014). As this agrees with historical placement (Linsley 1943), the tribal position of Townsendiellini seems settled, pending any future data. An intensive study of the bees of Pinnacles National Park (Messinger and Griswold 2003) yielded specimens of *Townsendiella* that ran to *Townsendiella pulchra* in the available revisionary study (Linsley 1943), but appeared to differ from other known *Townsendiella pulchra*. Similar unexpected variation was subsequently observed in specimens of *Townsendiella rufiventris*.

Here we describe a new species, *Townsendiella ensifera* sp. n., and discuss the taxonomic status of *Townsendiella rufiventris*. A key to known species of *Townsendiella*, using a combination of novel and historic characters, is also provided. Though few in species, this group may prove useful as indicator species (Sheffield et al. 2013). Comments are given for future research directions regarding the biogeography and evolution of bee host choice in this group.

## Materials and methods

A total of 443 specimens of *Townsendiella* from nine institutions were examined in this study, comprising both sexes of all four species (65 *Townsendiella ensifera*, 197 *Townsendiella pulchra*, 169 *Townsendiella rufiventris*, and 12 *Townsendiella californica*). The male genitalia of 12 *Townsendiella ensifera* and 12 *Townsendiella pulchra* were compared. Thorough examinations of maxillary palp morphology were also conducted (33 *Townsendiella ensifera*, 30 *Townsendiella pulchra*, 20 *Townsendiella rufiventris*, and 1 *Townsendiella californica*). A listing of specimens examined is provided as a supplementary file in reduced Darwin Core format. This file includes location descriptions for each specimen in the “locality” field, which when discussed throughout this text are given in double quotes.

Morphological terminology (e.g., T = tergum, S = sternum) follows Michener (2007), with two exceptions: the pseudopygidial area on T5 of *Townsendiella rufiventris* is called the lunule and the minute first maxillary palpomere of *Townsendiella rufiventris* is counted as a true palpal article rather than a tubercle (Linsley 1943). Images were taken with a Keyence VHF-500x Digital Microscope, and then processed using Photoshop CS5 Extended Version 12.0 (Adobe 2010, San Jose, CA). The map was generated with ArcMap10.2.2 (ESRI 2014, Redland, CA). All ecoregion calculations were based on the World Wildlife Fund (WWF) terrestrial ecoregions of the world (Olson et al. 2001).

Institutions that provided material, along with abbreviations used in the text and supplementary metadata, are as follows:

AMNH American Museum of Natural History, New York, NY, USA (J. Rozen, E. Wyman)

CAS California Academy of Sciences, San Francisco, CA, USA (N. Penny, B. Zuparko, V. Lee)

EMEC Essig Museum of Entomology, University of California, Berkeley, CA, USA (P. Oboyski)

NPIC U.S. National Pollinating Insects Collection, USDA-ARS Pollinating Insects Research Unit, Utah State University, Logan, UT, USA

SDNHM San Diego Natural History Museum, San Diego, CA, USA (J. Hung)

UCDC Bohart Museum of Entomology, University of California, Davis, CA, USA (L. Kimsey, S. Heydon)

UCR Entomology Research Museum, University of California, Riverside, CA, USA (D. Yanega)

UCSD University of California at San Diego, La Jolla, CA, USA (J. Hung)

USNM U.S. National Entomological Collection, National Museum of Natural History, Washington, D.C. (S. Brady, B. Harris)

## Results

**Figure 1.**
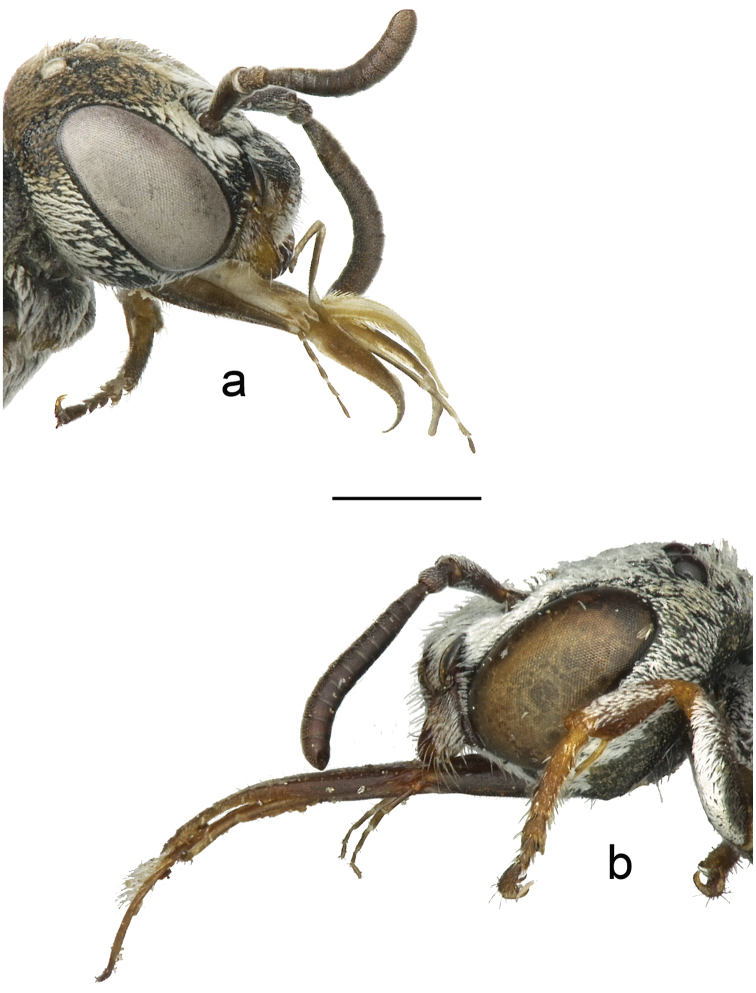
The male maxillary palp of **a** *Townsendiella ensifera* sp. n. (BBSL331567) and **b** *Townsendiella pulchra* (BBSL209492). The length of the terminal, fifth segment in *Townsendiella ensifera* sp. n. well exceeds that of the terminal, sixth segment in *Townsendiella pulchra*, likely due to the fusion of the fifth and sixth segments in the former. This character is reflected in both sexes. The scale bar represents 0.75 mm.

**Figure 2.**
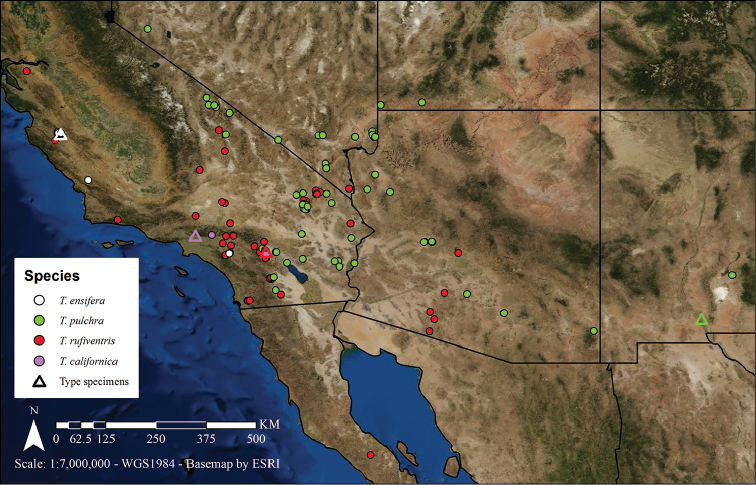
The distribution of *Townsendiella*. Species are color-coded as follows: *Townsendiella ensifera* sp. n. in white, *Townsendiella pulchra* in green, *Townsendiella rufiventris* in red, and *Townsendiella californica* in violet. The type locality for each species is given as a hollow triangle of its corresponding color.

Morphological analysis strongly supported the separation of the Pinnacles specimens from *Townsendiella pulchra*. Numerous characters which separate this species, *Townsendiella ensifera*, from other species of *Townsendiella* were identified. The number of palpomeres and their form in the maxillary palpus were found to be instrumental in distinguishing *Townsendiella ensifera* from the morphologically similar *Townsendiella pulchra* (Fig. 1). Both male and female specimens of *Townsendiella ensifera* were consistent in the characters of the maxillary palpus. In light of the utility of this character, spreading of the mouthparts is strongly encouraged in specimens of *Townsendiella*. Genitalic and other differences are further discussed in the subsequent key and species description. The geographic distribution of *Townsendiella ensifera* is also somewhat useful. Based on current records, *Townsendiella ensifera* is only known from California, in the South Coast Range and Transverse Range, ﻿apparently absent from much of the range of the similar *Townsendiella pulchra* (Fig. 2). Only *Townsendiella ensifera* and the dissimilar, more southerly *Townsendiella rufiventris* are currently known from the area north and west of the Transverse Range and Sierra Nevada. However, the presence of a single *Townsendiella ensifera* near Riverside, California demonstrates that this species is not limited to that region.

A DiscoverLife identification guide has also been created for this group. This guide may be accessed at: http://www.discoverlife.org/mp/20q?guide=Townsendiella

Instructions for the use of these guides are included within the Very Handy Bee Manual (Droege 2012).

### Key to the species of *Townsendiella*

**Table:** 

1	Metanotum not, or only slightly, produced medially, not produced into abrupt medial knob. **Female**: T5 without lunule (apical rim gradually and evenly concave; Fig. 3a). **Male**: gonoforceps only moderately flattened, opaque such that ventral spike is not visible in dorsal view; gonoforceps split apically, with long setae arising from tips	**2**
–	Metanotum with distinct, abrupt medial knob, clearly produced even if covered in thick pubescence. **Female**: T5 with apical lunule (depressed, finely-pitted, tessellate apicomedial region; Fig. 3b). **Male**: gonoforceps broadened and flattened, fully transparent such that ventral spike is visible in dorsal view; gonoforceps not split apically, with setae arising from tip short	***Townsendiella rufiventris* Linsley, 1942**
2	Marginal cell elongate, about equal in length to distance from tip of marginal cell to wing tip or longer; posterior margin of first submarginal cell longer than posterior margin of second submarginal cell, but clearly less than twice length of posterior margin of second submarginal cell; second submarginal cell with distal vein strongly curved, much longer than proximal vein; apical fasciae of T2–T4 incompletely divided medially or, when completely divided, only narrowly so. **Female**: mesoscutellum black	**3**
–	Marginal cell shorter, length less than that from tip of marginal cell to wing tip; posterior margin of first submarginal cell about twice as long as posterior margin of second submarginal cell, sometimes slightly shorter; second submarginal cell with distal vein only slightly curved, approximately same length as proximal vein; apical fasciae of T2–T4 strongly interrupted medially. **Female**: mesoscutellum red	***Townsendiella californica* Michener, 1936**
3	Maxillary palp with five palpomeres, terminal palpomere about equal in length to long second palpomere (Fig. 1a). **Female**: S5 with setae evenly covering segment throughout (may be worn off in older specimens); pygidial plate relatively flat and smooth, punctures distinct. **Male**: genital capsule in dorsal view with upper gonostylus small and short, distance from tip of upper gonostylus to tip of lower gonocoxite equal to twice maximum width of upper gonostylus or greater (Fig. 4a)	***Townsendiella ensifera* sp. n.**
–	Maxillary palp with six palpomeres, terminal palpomere clearly shorter than long second palpomere, usually about equal to fifth palpomere (Fig. 1b). **Female**: S5 with apicomedial, asetose V- shaped patch, defined by slight integumental indent; pygidial plate craggy, punctures indistinct. **Male**: genital capsule in dorsal view with upper gonostylus large and long, distance from tip of gonostylus to tip of gonocoxite equal to about maximum width of upper gonostylus or less (rarely greater) (Fig. 4b)	***Townsendiella pulchra* Crawford, 1916**

### Townsendiella ensifera sp. n.

Taxon classificationAnimaliaHymenopteraApidae

http://zoobank.org/D80DEF1E-DFC2-4D3F-A539-1FC439A78A67

**Figure 3.**
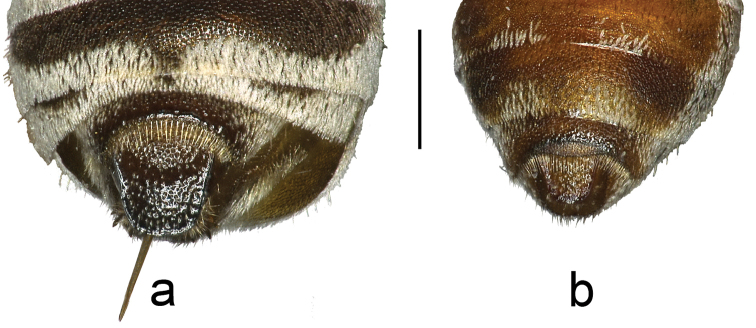
The female T5 of **a** *Townsendiella ensifera* sp. n. (BBSL331869) and **b** *Townsendiella rufiventris* (BBSL209499). The latter has a distinctly impressed, finely-pitted, and tessellate lunule medially in the pseudopygidial area, a feature unique within *Townsendiella*. The scale bar represents 0.5 mm.

**Figure 4.**
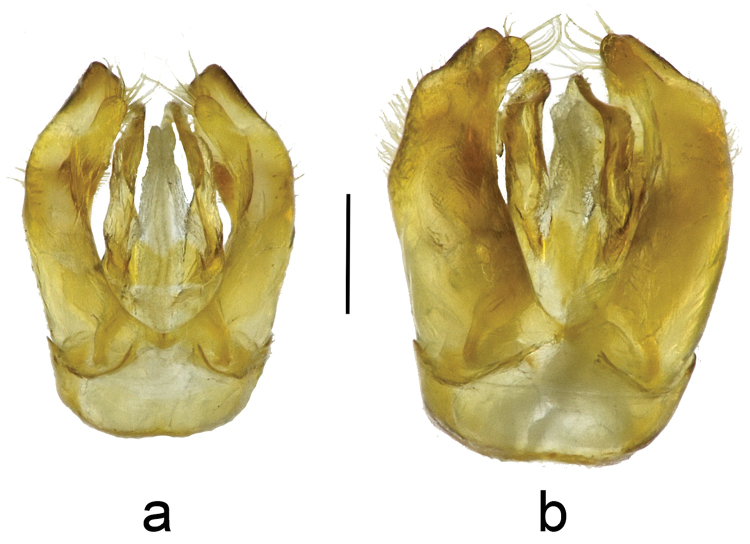
The male genitalia of **a** *Townsendiella ensifera* sp. n. (BBSL259479) and **b** *Townsendiella pulchra* (BBL340294). Dorsal view of genital capsule and gonostylus. The scale bar represents 0.25 mm.

**Figure 5.**
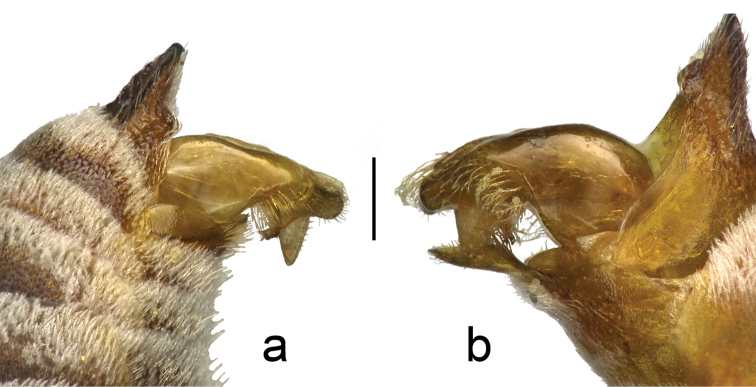
The male genitalia of **a** *Townsendiella ensifera* sp. n. (BBSL259415) and **b** *Townsendiella pulchra* (BBSL349670). Lateral view of gonocoxite, with focus on setae. The scale bar represents 0.25 mm.

**Figures 6–8.**
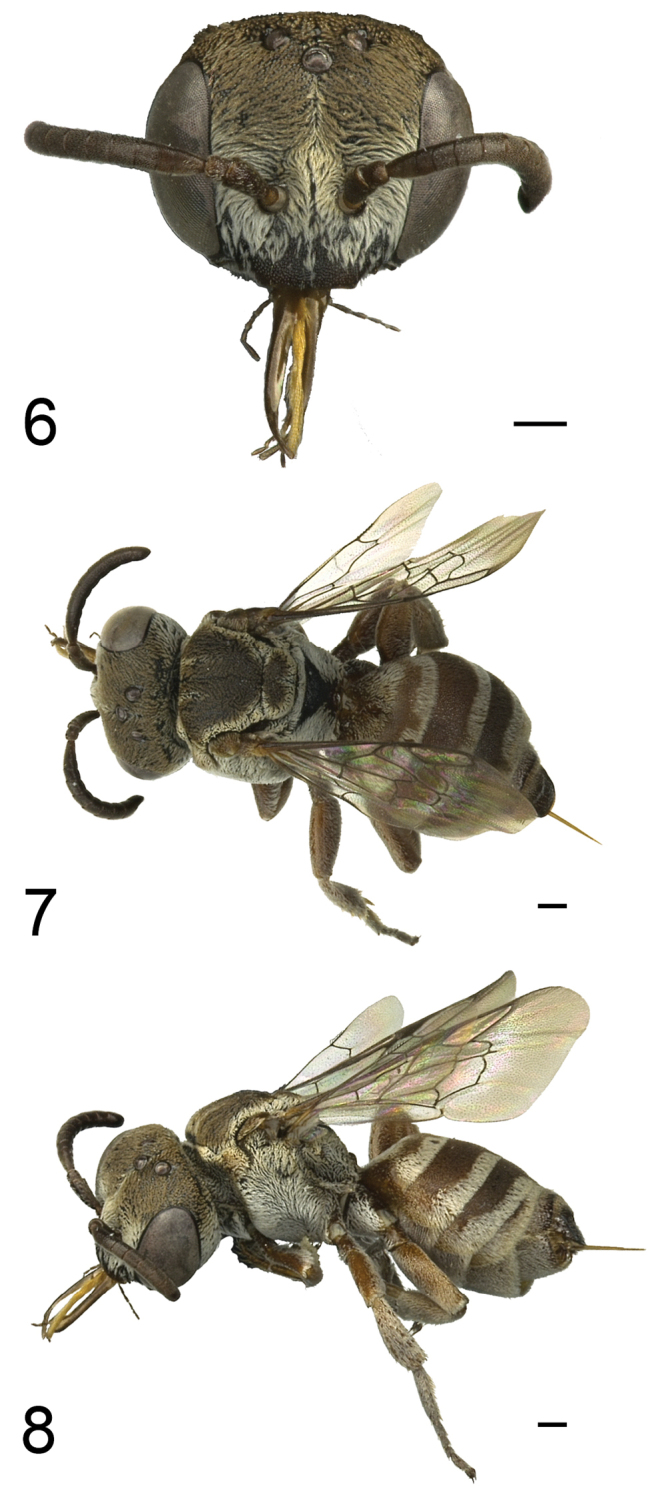
General appearance of the female *Townsendiella ensifera* sp. n. holotype (BBSL331869). **6** Anterior view of face **7** Dorsal view of body **8** Lateral view of body. All scale bars represent 0.25 mm.

Figs 1a , 3a , 4a , 5a , and 6–8

#### Type-locality.

USA, California: San Benito County, Pinnacles National Park, East of Mount Defiance, 36.46060 -121.15210, Blue oak woodland, white pantrap, 29 May 1999, O. Messinger leg., host unknown.

#### Holotype.

Female, pinned. Original label: “USA CA San Benito Co., / Pinnacles Natl. Mon., / East of Mount Defiance / 36°28.24’N 121°09.13’W [white typed label]” “White pantrap, burn / Blue oak woodland [white typed label]” “29 May, 1999 / O. Messinger [white typed label]” “NativeBeeSurvey / USDA,Logan,Utah / BBSL331869 [barcode label].”

#### Paratypes.

Nine topo-typical specimens (1F8M); all deposited in the NPIC. Unique specimen identifiers are as follows: BBSL330902, BBSL331852, BBSL331878, BBSL331886, BBSL331887, BBSL331891, BBSL332126, BBSL332137, and BBSL332139. Selected specimen data for each paratype is available in the Suppl. material 1.

#### Other material.

Additional records are detailed in the Suppl. material 1.

#### Diagnosis.

Both males and females are most similar to *Townsendiella pulchra* and are separated easily from other *Townsendiella* by the absence of a medially projecting knob on the metanotum, the length of the marginal cell being about equal or greater than the distance from its posterior tip to the posterior tip of the wing, and the asymmetrical second submarginal cell with the longer, more curved distal vein. From *Townsendiella pulchra*, it may be differentiated by the following characteristics: five maxillary palpomeres, the final palpomere almost as long as the second palpomere; male pygidial plate narrower, sharper at the tip; female S5 lacking a pubescent apicomedial area, setal density roughly even along rim; when viewing the genital capsule laid flat against a surface, with your view perpendicular to the surface: the tip of the male gonostylus is about its maximum width distant from the gonocoxite tip, while in *Townsendiella pulchra* it is almost equal; male gonocoxite with shorter, fewer, and less plumose setae present ventrolaterally (Fig. 5a) compared to *Townsendiella pulchra* (Fig. 5b); and male genital capsule smaller overall, with gonocoxites less expanded apically (Figs 4, 5).

#### Description.

**Female**: Head: Pubescence dense, white, covering one-half to three-fourths of height of compound eye, becoming sparser, less plumose, and often off-white in coloration dorsally. Facial integument black, reddening, if present, limited to labrum and rim of clypeus. Punctation relatively dense throughout, integument shining between punctures. Mandible simple, basal three-fourths to four-fifths of integument dull orange to bright yellow, tip reddish-brown. Clypeus protuberant from anterior margin of compound eye by one-third to one-half of maximum compound eye width. Integument of antenna dark brown to black, sometimes light brown. Compound eyes slightly converging ventrally. Lateral ocellus distinctly closer to rear margin of head than compound eye, separated from rear margin by roughly 2–2.5× lateral ocellar diameter.

Mesosoma: Pubescence all white except for light brown areas of mesoscutum and mesoscutellum. Pubescence dense over mesosoma, except slightly less dense where brown, sparser on pronotum anteriorly, mesepisternum anteriorly, and propodeal triangle below metanotum. Mesoscutum with pubescence primarily light brown except along border, with two thin, longitudinal stripes of white setae intruding posteriorly from anterior margin for one-third to one-half of mesoscutal length. Mesoscutellum with pubescence largely light brown, bordered by white, with anterior-directed stripe of white setae from posterior midline. Integument dark brown to black, often with pronotal lobe reddened, less commonly reddened ventrally or elsewhere on pronotum. Punctation dense throughout mesosoma, except propodeal enclosure where absent or obscured by tessellation, integument otherwise relatively smooth and shiny. Tegula brown, slightly transparent, but obscured by dense setae.

Wings: Wings equal to 3.0–3.1× medial length of mesoscutum along longitudinal axis. Length of marginal cell slightly greater than distance from distal tip of marginal cell to apical tip of wing. Length of posterior margin of first submarginal cell greater than that of second, but clearly less than twice length of second. 2m-cu usually interstitial with juncture between first and second submarginal cells, or only slightly past this point, creating four-way intersection.

Legs: Integumental color variable, ranging from dark brown to light reddish-brown; tarsi typically black. White pubescence present ventrally on femora, variable in extent. Outer surfaces of tibiae densely clothed in white pubescence, densest on metatibia. Thicker, spine-like setae readily apparent on meso- and metatibiae, usually obscured by pubescence on protibia.

Metasoma: Pubescence white except in basal areas of terga, where it is slightly browned; brown setae obscured on T5 by white setae throughout. Pubescence denser and more branched apically on terga, creating distinct setal bands on T1–T4, each of which is usually thinner medially and thicker laterally, with V-shaped medial notch. Sternal pubescence primarily white but thinner and sparser in basal and lateral areas, appearing apically banded at some angles. S5 pubescence relatively even throughout. Integumental color of terga highly variable, ranging from nearly all black to nearly all red. T5 even across its rim, with simple setae extending posteriorly. Pygidial plate roughly square, only slighter wider at base than apex due to rounded posterior corners; entire rim slightly raised into carina, marked by darker, blackish integument there compared to pitted, redder interior.

**Male** similar to female, except as follows: Head: Pubescence all white, dense over much of lower face up to slightly below top of compound eye and on gena to about top of compound eyes, becoming less branched and, in some specimens, slightly off-white near vertex. Clypeus protuberant from anterior margin of compound eye by about half of compound eye width or slightly less. Paraocular area raised into flange adjacent to clypeus, this area impunctate and shiny. Integument of antenna dark brown to black, sometimes slightly lighter brown on apical segments.

Mesosoma: Integument dark brown to black, rarely with dark reddish-brown coloration on pronotum or venter.

Wings: Wings equal to 3.1–3.2× medial length of mesoscutum along longitudinal axis.

Legs: Integumental color variable, ranging from dark brown to light reddish-brown. Metabasitarsus and metatarsus more obscured by white, plumose setae than on other legs.

Metasoma: Pubescence white except in basal areas of terga, where slightly browned; brown setae intermixed with lighter setae in basal area of T6. Pubescence denser and more branched apically on terga, creating distinct setal bands on T1–T4, each of which is usually thinner medially and thicker laterally, typically with those of T5 and T6 thicker, more uniform overall. S6 pubescence notably thinner than preceding sterna, not appearing apically banded. Integumental color of terga variable from dull reddish-brown to near black, more often black. Pygidial plate coming to acute point, sides roughly straight to weakly convex, heavily-sculpted medial region often raised, integumental color darker brownish around rim and reddened interiorly.

#### Etymology.

The specific epithet, *ensifera*, is Latin for sword-bearing. This name is primarily a reference to the elongate, sword-shaped terminal maxillary palpomere, and secondarily a reference to its cleptoparasitic life history.

#### Distribution.

*Townsendiella ensifera* is known primarily from the South Coast Range of California, where it has been found at two localities: Pinnacles National Park in San Benito County and San Luis Obispo County, 6 mi NE Santa Margarita. The only other record is a single specimen from south of the Transverse Range (“The Gavilan”) near Riverside, California. More collections are necessary to determine the true extent of this species’ range, although it presently appears more restricted than most other *Townsendiella*.

#### Phenology.

Known to be active from early May to late August. Within Pinnacles National Park, where multiple collections took place, it was collected from early May to early July.

#### Bee hosts.

There is no direct knowledge concerning the host of *Townsendiella ensifera*. Given the apparent preference of the similar *Townsendiella pulchra* for species of *Hesperapis* Cockerell and the extensive sampling of Pinnacles National Park, *Hesperapis* as a potential host may be inferred as a working hypothesis. Only two species of *Hesperapis*, Hesperapis (Amblyapis) ilicifoliae (Cockerell, 1910) and Hesperapis (Panurgomia) regularis (Cresson, 1878), have been recorded from Pinnacles. *Hesperapis regularis* is many times the body size (>12 mm in length) of *Townsendiella ensifera*, while *Hesperapis ilicifoliae* is about 5–6 mm in length, suggesting *Hesperapis ilicifoliae* is the better candidate as host. The likelihood of *Hesperapis ilicifoliae* as host increases when considering that it belongs to the same subgenus as Hesperapis (Amblyapis) larreae Cockerell, 1907, the host for *Townsendiella pulchra* (Rozen and McGinley 1991). Further support is derived from the synchrony of their flight periods; both *Hesperapis ilicifoliae* and *Townsendiella ensifera* fly from May to July, with the majority of records from June and July. It should also be noted that the specimen of *Townsendiella ensifera* from Gavilan, California was collected on *Adenostoma fasciculatum* Hook. & Arn., the plant which *Hesperapis ilicifoliae* specializes on, and in an area where *Hesperapis ilicifoliae* has been collected previously (Moldenke and Neff 1974, DiscoverLife 2014). In fact, *Townsendiella ensifera* has only been collected in areas where *Hesperapis ilicifoliae* is known. Based on all evidence, *Hesperapis ilicifoliae* is currently the most likely host for *Townsendiella ensifera*.

#### Floral hosts.

Polygonaceae: *Eriogonum fasciculatum* Benth. and Rosaceae: *Adenostoma fasciculatum*.

#### Discussion.

This species is much closer in body form to *Townsendiella pulchra* than the other species are to each other or to the pair of *Townsendiella ensifera* and *Townsendiella pulchra*. It is possible that these very distinct species led to a general expectation of great differentiation between species in the group and that the relatively minor differences between *Townsendiella ensifera* and *Townsendiella pulchra* were overlooked as a result.

### Townsendiella californica

Taxon classificationAnimaliaHymenopteraApidae

Michener, 1936

#### Holotype.

female, pinned; Altadena, California; 6-26-35 [26 June 1935]; deposited in CAS (Type#4544).

#### Diagnosis.

This species is immediately separable from all other species by its wing venation. The marginal cell is the shortest of any *Townsendiella*, its maximum length significantly shorter than the distance from its apical tip to the apex of the wing. The posterior margin of the first submarginal cell is roughly twice the length of the second submarginal’s, while in other species the first submarginal cell is closer to 1.5× the length of the second. The second submarginal cell forms a nearly symmetrical triangle with the distal vein nearly straight and nearly the same length as the proximal vein.

#### Distribution.

*Townsendiella californica* has a relatively restricted range in comparison to the other species of the genus. It is currently known from localities along the southern edge of the Transverse Range and north of the nearby Mt. San Jacinto. Interestingly, these collections are all along the edge of the California montane chaparral and woodlands ecoregion. It may be that this species, its host, or both inhabit a very narrow ecological niche.

#### Phenology.

The phenology of this species is difficult to ascertain due to few collection records, though it appears to be active from late April through June.

#### Bee hosts.

Uncertain. This species is hypothesized to be cleptoparasitic on Hesperapis (Zacesta) rufipes (Ashmead, 1899) based on observation of *Townsendiella californica* flying over a nesting aggregation of the former (Michener 1936). Multiple attempts were made to confirm the host at the same site; the population has apparently been extirpated by urban sprawl from the Altadena, California area (Stage 1966).

#### Floral hosts.

No floral records are known for this species.

#### Discussion.

This exceptionally rare species is known from the fewest specimens of any *Townsendiella*. As such, much remains to be discovered regarding its distribution, host specificity, and environmental constraints.

### Townsendiella pulchra

Taxon classificationAnimaliaHymenopteraApidae

Crawford, 1916

Figs 1b , 4b , and 5b

#### Holotype.

female, pinned; Las Cruces, New Mexico; 5.12 [12 May]; deposited in USNM (Type#20831).

#### Diagnosis.

This species may be distinguished from *Townsendiella californica* and *Townsendiella rufiventris* by the combination of the following characteristics: long marginal cell, the maximum length of which is about equal to or longer than the distance from the marginal cell tip to the apex of the wing; metanotum lacking medial projection, only very gradually curved throughout its width; and the female lacking a lunule on T5. *Townsendiella pulchra* is more similar to *Townsendiella ensifera* than to the other species, and may be separated from it as presented in the latter’s species account.

#### Distribution.

Present from west-central Nevada to central New Mexico, and ranging southward to the Mexican border, *Townsendiella pulchra* has arguably the largest range in the genus. With both the most northerly and easterly collection sites, *Townsendiella pulchra* inhabits a number of different ecoregions, given here in order from most to least collection localities: Mojave Desert, Sonoran Desert, Chihuahuan Desert, Great Basin shrub steppe, and Colorado Plateau shrublands.

#### Phenology.

*Townsendiella pulchra* has been collected primarily during April and May, although a small number of records exist from late March and June. Surprisingly, there are two records of this species from August near Portal, Arizona. It may be that this species is active during the fall in the Chihuahuan Desert, in time with monsoonal rains. Additional collections are necessary to investigate this possibility, though the collection of the Las Cruces type specimen in May demonstrates it does not fly exclusively in fall in the Chihuahuan Desert.

#### Bee hosts.

This species is known to invade and oviposit within nests of Hesperapis (Amblyapis) larreae, mistakenly placed in Hesperapis (Panurgomia) in previous host associations (Michener 1936, Michener 2000, Rozen and McGinley 1991). The description of this behavior includes extensive notes on the interactions between these two species (Rozen and McGinley 1991).

#### Floral hosts.

Asteraceae: *Baileya pleniradiata* Harv. & A. Gray, *Baileya* sp. Harv. & A. Gray ex Torr., *Chaenactis* sp. DC.; Boraginaceae: *Tiquilia hispidissima* (Torr. & A. Gray) A.T. Richardson; Fabaceae: *Psorothamnus arborescens* (Torr. ex A. Gray) Barneby, *Psorothamnus fremontii* (Torr. ex A. Gray) Barneby, *Psorothamnus schottii* (Torr.) Barneby, *Psorothamnus* sp. Rydb.; Zygophyllaceae: *Larrea tridentata* (DC.) Coville.

#### Discussion.

This species is the best known of the *Townsendiella*, given the extensive life history work conducted on it and its host (Rozen and McGinley 1991). It will be interesting to see if all *Townsendiella* share similar life histories, once such information becomes available for the remaining species.

### Townsendiella rufiventris

Taxon classificationAnimaliaHymenopteraApidae

Linsley, 1942

Fig. 3b

#### Holotype.

female, pinned; Palm Springs, California; Mar 26, 1932; deposited in CAS (Type#14881).

#### Diagnosis.

The female of this species may immediately be separated from the other *Townsendiella* by the presence of the lunule on T5 (apicomedial impressed rim with dense, fine punctures). Both females and males also have a strong medial production on the metanotum, which is not seen in other species. The male gonoforceps are quite distinctly flattened and relatively transparent, lacking a distinct gonostylus.

#### Distribution.

The distribution of *Townsendiella rufiventris* is exceptionally broad among the *Townsendiella*, spanning from Baja California, Mexico, and the eastern Sonoran Desert, extending northward through the coastal ranges nearly to the San Francisco Bay. As such, this species has both the most southerly and westerly collection events of any *Townsendiella*. It inhabits several ecoregions, given here in order of most to least collection localities: Mojave Desert, Sonoran Desert, California coastal sage and chaparral, California interior chaparral and woodlands, California montane chaparral and woodlands, and Baja California Desert.

#### Phenology.

This species is known to fly from mid-March through July, although its phenology appears to differ throughout its range. Within the Mojave, it appears to be most active from late March through May. In the South Coast Range, however, it appears to be active in June and July. Further collections from its northern distribution are necessary to test this possibility.

#### Bee hosts.

Interestingly, *Townsendiella rufiventris* appears to use the halictid genus *Conanthalictus* Cockerell as hosts, although prior publications have not listed hosts at the species-level (Linsley 1958, Rozen and McGinley 1991). A determination label by the late Paul D. Hurd with a date of 1963 from the Essig Museum gives the determination of *Townsendiella rufiventris* and states it was “flying about nest site of *Conanthalictus nigricans* Timb.” The label was placed before a series of *Townsendiella rufiventris* from “San Marcos Ranch HQ, Santa Inez Mts,” found near Santa Barbara Co., California. More recently, *Townsendiella rufiventris* has been collected northwest of San Bernardino, California (“N Sierra Ave”) invading the nests of *Conanthalictus bakeri* Crawford, 1907 (D. Yanega, unpublished observations, 30 April 2015). Circumstantial evidence has also been found in the association of high numbers of *Townsendiella rufiventris* at sites with *Conanthalictus bakeri* (“Jamul CA” and “Spring Valley CA”) and *Conanthalictus wilmattae* Cockerell, 1936 (“Anza-Borrego, In-Ko-Pah Park” and “Anza-Borrego: Peña Spring”), all sites which yielded few to no *Hesperapis*, though no positive host associations were possible (J. Hung, unpublished observations, 14 May 2015).

#### Floral hosts.

Asteraceae: *Lasthenia californica* DC. ex Lindl.; Boraginaceae: *Cryptantha intermedia* (A. Gray) Greene, *Cryptantha* sp. Lehm. ex G. Don, *Phacelia distans* Benth., *Phacelia* sp. Juss.; Onagraceae: *Chylismia munzii* (P.H. Raven) W.L. Wagner & Hoch.

#### Discussion.

The possibility that *Townsendiella rufiventris* is two species was explored based on observations by Doug Yanega (pers. comm., 10 December 2013). The primary character investigated was the form of the lunule on the female T5, a finely-pitted, tessellate apicomedial depression filling the otherwise concave rim, the presence of which is a unique character for *Townsendiella rufiventris*. The species may be roughly split into two series, those with a flat apical rim on the lunule (series 1) and those with an apical lunule which projects farthest medially (series 2; allied with type of *Townsendiella rufiventris*). However, the reliability of the lunule as a character is questionable in light of its apparent flexibility, demonstrated by the variability in its angle relative to the rest of T5 across specimens of the same series. The area basal to the lunule is also variable, going from sparsely pitted and shiny in series 1 to densely, craggily pitted and dull in series 2, although numerous exceptions have been discovered. The proportion of black integument basal to the lunule also varies, with more in series 1 and less in series 2, but exceptions to this have also been found. The male genitalia were also examined, using four males collected with females of series 1 and three specimens associated with series 2 females, but no diagnostic characters were detected. No characters from either sex which clearly and consistently delineate the two entities were discovered. A geographic split is also impossible; although series 2 is primarily found in southern California, the range of series 1 appears to completely envelop that of series 2. It must also be noted that females of both series 1 and series 2 were found from the same collection event thrice, casting further doubt on the existence of two species.

## Discussion

It is now clear that the *Townsendiella* from Pinnacles National Park (*Townsendiella ensifera*) represent a species separate from *Townsendiella pulchra*. Consistent differences of the mouthparts, male genitalia, and other characters confirm this. The apparently allopatric distribution of these species further supports this distinction. Despite intense collection effort near Riverside, California, by P.H. Timberlake and others, only a single male of *Townsendiella ensifera* was collected (Fig. 2); no *Townsendiella pulchra* were detected. The closest record of *Townsendiella pulchra* is only 75 kilometers away, but this seemingly insignificant distance represents the transition from the California coastal sage and chaparral ecoregion to the northeastern limit of the Sonoran Desert ecoregion, a significant ecological leap.

The question of potential cryptic species within *Townsendiella rufiventris* warrants further investigation. It is possible that there may be two or more species, which would explain the high level of variation and the exceptions to the character patterns discovered. Given this high level of variation, there is inadequate material available at this time to determine whether or not *Townsendiella rufiventris* is a species complex. Future study using molecular techniques would be beneficial, but access to molecular-grade specimens is limited due to the rarity of this group. Although Michener (2000) previously rejected the subgenera of *Townsendiella*, hesitantly doing so as they were “unnecessary” in light of so few species, they may prove useful if *Townsendiella rufiventris* is found to be a species complex.

The biogeography and host evolution of *Townsendiella* are areas ripe for research. Although all species are found in the southwestern US and adjacent Mexico, the seemingly disjoint distributions of *Townsendiella ensifera* and *Townsendiella rufiventris* raise the questions of how and when they arrived in the South Coast Range. It may be that ancestral populations had much larger distributions, including the deserts and Mediterranean California, but were then separated by Neogene uplift or Pleistocene climate change, as they have apparently played a role in the diversification of other Hymenoptera (Wilson and Pitts 2010). An alternative explanation is dispersal, where both species have surmounted the incomplete barrier imposed by the Transverse Range and lower Sierra Nevada to become established in Mediterranean California. As Mediterranean vegetation occurs along the sides of the Transverse Range and patchily throughout it, it may be that avenues of dispersal are even currently available to this group. The directionality of dispersal under the latter hypothesis is another open question to be investigated.

Host evolution may prove to be an even more interesting area of research. Most host records are in the melittid genus *Hesperapis*, but *Townsendiella rufiventris* apparently attacks *Conanthalictus*. The most obvious question is how this host switch occurred. As *Townsendiella* is known to search for nesting sites, and oligoleges often nest near their host plants, it may be that the switch was facilitated by both *Conanthalictus* and *Hesperapis* nesting near a shared host plant (Rozen and McGinley 1991). Two possibilities are the plant genera *Nama* L. and *Phacelia* Juss., on both of which some *Conanthalictus* and *Hesperapis* specialize (Moldenke and Neff 1974, Rozen 1987, Stage 1966). Given that *Conanthalictus bakeri*, *Conanthalictus nigricans* Timberlake, 1961, and *Conanthalictus wilmattae* are all specialists on *Phacelia*, the latter possibility seems more likely at present.

A second, related question is why only *Townsendiella rufiventris* possesses a lunule. As this species appears to be the only *Townsendiella* which attacks *Conanthalictus* nests, it may be that the lunule serves a special function for invading or ovipositing within the nests of that group. As *Townsendiella pulchra* apparently enters open nest cells, as evidenced by their oviposition into the cell wall (Rozen and McGinley 1991), there are many ways in which the female could be using the lunule, such as smoothing soil or applying secretions. More work is necessary to understand the evolution of these elusive bees.

## Supplementary Material

XML Treatment for Townsendiella ensifera

XML Treatment for Townsendiella californica

XML Treatment for Townsendiella pulchra

XML Treatment for Townsendiella rufiventris

## Supplementary materials

Supplementary material 1 *Townsendiella* specimen data Data type: species data This dataset is made available under the Open Database License (http://opendatacommons.org/licenses/odbl/1.0/). The Open Database License (ODbL) is a license agreement intended to allow users to freely share, modify, and use this Dataset while maintaining this same freedom for others, provided that the original source and author(s) are credited.Michael C. Orr, Terry L. Griswold
